# Examining the hospital costs of children born into relative deprivation in England

**DOI:** 10.1136/jech-2023-221175

**Published:** 2024-05-15

**Authors:** Veronica Dale, Nils Gutacker, Jonathan Bradshaw, Karen Bloor

**Affiliations:** 1 Health Sciences, University of York, York, UK; 2 Centre for Health Economics, University of York, York, UK; 3 Social Policy Research Unit, University of York, York, UK

**Keywords:** POVERTY, CHILD HEALTH, COHORT STUDIES

## Abstract

**Objective:**

To examine the association between being born into relative deprivation and hospital costs during childhood.

**Design:**

Retrospective cohort study.

**Methods:**

We created a birth cohort using Hospital Episode Statistics for children born in NHS hospitals in 2003/2004. The Index of Multiple Deprivation (IMD) rank at birth was missing from 75% of the baby records, so we linked mother and baby records to obtain the IMD decile from the mother’s record. We aggregated and costed each child’s hospital inpatient admissions, and outpatient and emergency department (ED) attendances up to 15 years of age. We used 2019/2020 NHS tariffs to assign costs. We constructed an additional cohort, all children born in 2013/2014, to explore any changes over time, comparing the utilisation and costs up to 5 years of age.

**Results:**

Our main cohort comprised 567 347 babies born in 2003/2004, of which we could include 91%. Up to the age of 15 years, children born into the most deprived areas used more hospital services than those born in the least deprived, reflected in higher costs of inpatient, outpatient and ED care. The highest costs and greatest differences are in the year following birth. Comparing this with the later cohort (up to age 5 years), the average cost per child increased across all deprivation deciles, but differences between the most and least deprived deciles appeared to narrow slightly.

**Conclusions:**

Healthcare utilisation and costs are consistently higher for children who are born into the most deprived areas compared with the least.

**WHAT IS ALREADY KNOWN ON THIS TOPIC**

- Deprivation is associated with poorer health in childhood, but the association with healthcare utilisation is less known. This study uses NHS hospital data to examine the impact of relative deprivation on utilisation and costs of healthcare services throughout childhood.

**WHAT THIS STUDY ADDS**

- Hospital utilisation and associated costs were consistently higher for the children born into more deprived areas over the first 15 years of life.

**HOW THIS STUDY MIGHT AFFECT RESEARCH, PRACTICE OR POLICY**

- This study highlights the differential use of hospital services for children born into relative deprivation and their higher costs, confirming the importance of addressing inequalities at all levels of policy and practice.

## Introduction

Children living in areas of relative deprivation face increased risks of poorer health and other adverse life consequences.^1^ The period of early childhood is increasingly recognised as a critical period of development and the most highly sensitive to external influences.^1 2^ Children living in areas of relative deprivation face many increased health risks associated with poverty (which means that children are more likely to die in the first year of life, be born small, be bottle fed, breathe secondhand smoke, become overweight, suffer from asthma, have tooth decay and die in an accident^3^). They may also face difficulties in accessing healthcare.^4^ Household-level material deprivation restricts parents’ ability to buy goods and services that can enhance children’s development and also exposes families to environmental stressors which can affect infants’ brain development.^5 6^ Through these and no doubt other mechanisms, relative deprivation has been demonstrated to affect longer-term health, including links with inflammatory conditions and cardiovascular disease.^7^ Health and care professionals treat the consequences of poverty and deprivation, and the health effects of childhood deprivation may result in a significant burden on health systems.

Most studies on the costs and use of healthcare services by different socioeconomic groups have been cross-sectional; consideration of longitudinal relationships is much less common. A recent paper^8^ studying lifetime inpatient hospital costs in England by level of neighbourhood deprivation found a social gradient in both current and lifetime hospital costs, with an overall estimate that socioeconomic inequality cost the NHS £4.8 billion in 2011/2012 as a result of excess hospital admissions. A report on emergency hospital use for children and young people^9^ explored emergency admissions and emergency department (ED) attendances by deprivation quintile in England, comparing 2005/2006 with 2015/2016. We are not aware of existing national studies on the impact of deprivation on utilisation and cost of healthcare services throughout childhood. In this paper, we aim to explore, using a longitudinal cohort approach, whether children born in England in areas of relative deprivation use hospital care differently over the course of their childhood and how much any differences cost. As well as informing policy about the health and fiscal consequences of deprivation, this could provide a method for assessing trends over time and any associations with national or regional policy interventions.

## Methods

### Study design and data sources

This is a retrospective cohort study, following guidance from the Reporting of studies Conducted using Observational Routinely Collected Data guidelines.^10^ We examine the association between being born into an area of relative deprivation and the costs of hospital utilisation up to the age of 15 years, using Hospital Episode Statistics (HES). HES records contain information on hospital activity in England;^11^ these records include a score and rank of relative deprivation (Index of Multiple Deprivation, IMD)^12^ for every small area or neighbourhood (lower-layer super output area, LSOA). This provides a measure of the relative level of deprivation of 32 844 small areas in England.

### Population

We constructed a birth cohort for babies born between 1 April 2003 and 31 March 2004 using HES birth records. An additional cohort for babies born between 1 April 2013 and 31 March 2014 provides a comparison of hospital utilisation up to age 5 years.

Prior to the financial year 2003/2004, the recording of babies’ NHS numbers in birth records was incomplete, which created problems in linking birth records with subsequent hospital admissions. The implementation of NHS Numbers for Babies in October 2002 led to improvements in completeness and an increase in the number of birth and hospital admission records that could be linked.^13^ Choosing a cohort from 2003/2004 maximises the number of complete records available while still allowing for a follow-up of 15 years.

Prior to 2012/2013, postcodes were not routinely extracted for birth records, meaning that the completeness of the IMD rank in the baby records was low; however, it was present in 99% of the mothers’ records.^14^ To maximise the number of baby records with a valid IMD rank, we linked mother and baby records to obtain the IMD from the mother’s record for the baby.

### Linkage

We linked the mother and baby records to each other following methods of deterministic linking followed by probabilistic linking, using the variables, cut-off and methods outlined by Harron and colleagues.^15^

Deterministic linking requires agreement on a set of common identifiers, although some missing variables were allowed. Each baby and mother record had to match on at least three of the six linking variables (general practitioner (GP) practice, maternal age, birth weight, gestation, birth order and sex) and not disagree on any. In addition, the mother’s admission date had to be within 30 days before and 7 days after the baby’s birth date, and the provider code (hospital) also had to match.

Probabilistic linking uses frequency-based match weights so that the level of agreement can vary depending on the value of the variables, for example, dates that are closer would have a higher level of agreement than those further apart. Positive weights indicate the level of agreement, and a negative weight indicates disagreement. Weights are calculated for each matching variable and then summed to create an overall match weight. To reduce the number of comparison pairs, mother and baby records were only considered if they were from the same hospital and the estimated delivery dates from mother and baby were within 30 days of each other. We used descriptive statistics and manually checked random samples of the linked records with different match weights to check that the linking process had worked. Further description of the linking variables is in the online supplemental appendix.

10.1136/jech-2023-221175.supp1 Supplementary data

### Cohorts

We identified 567 347 unique birth episodes in the year 2003/2004. Only 25% of baby records included their mothers’ place of residence at the time of the birth, and therefore their IMD rank, but this was present in 99% of the mothers’ records. Deterministic linkage successfully linked 48% of baby records to a maternal record with IMD recorded. A further 37% were probabilistically linked, with the IMD recorded, and further 6% of baby records that had not been linked had the IMD already recorded. In total, a valid IMD was obtained for 515 889 babies, 91% of the birth cohort.

An additional cohort was created for babies born between 1 April 2013 and 31 March 2014 to compare utilisation over time up to age 5 years. For the 2013/2014 cohort, the completeness in the baby records for IMD rank was much higher, at 82%. Over time, the quality and completeness of HES data have improved.^13^ This meant for the 2013 cohort deterministic linkage was more successful—more mother and baby records were linked (65%) than the 2003 cohort. Using these linked records and those with IMD recorded directly resulted in 576 624 baby records with a valid IMD, 91% of the birth cohort. Further details of the completeness of the data in each cohort are included in the online supplemental appendix.

### Outcomes

The primary outcome of the analysis was the health utilisation costs up to the age of 15 years for outpatient attendance and hospital inpatient episodes for children born between 1 April 2003 and 31 March 2004.

HES ED data are only available from 2007. As it is missing from the first 4 years of data for the 2003 cohort, the cost of ED attendances is presented from 4 to 15 years of age.

Secondary outcomes were the costs of outpatient attendance and hospital inpatient episodes up to age 5 years. Comparisons were made between the cohort born in 2003/2004 and a cohort born 10 years later in 2013/2014.

### Analysis

The number of hospital inpatient episodes, outpatient attendances and ED attendances were collated for each baby record up to age 15 years. We excluded the birth episode from our calculations since they occurred for all babies in our dataset (ie, this was a constant). To enable comparison between different cohorts, the time was converted to years since birth. The NHS tariff cost per attendance is determined by the healthcare resource group (HRG) to which each patient is assigned. However, both the HRG system and their NHS tariffs changed throughout the time series, which complicates longitudinal analysis. To ensure consistency across time, we first calculated the average cost to the NHS of, for example, an outpatient attendance for all children using outpatient services between April 2019 and March 2020 using the 2019/2020 NHS tariff and event rates. We then assigned this cost estimate to all relevant attendances in our dataset. For hospital inpatient episodes, we calculated average costs by HRG chapter (the first letter of the HRG code), age, sex and admission type (elective, emergency or other) to better differentiate across activities with different resource requirements. For a small proportion of hospital admissions, average costs could not be calculated because the 2019/2020 NHS tariff did not assign prices to HRGs in that chapter. In this case, costs were imputed as the NHS tariff for HRG PX57C (paediatric examination, follow-up, special screening or other admission, with comorbidity or complexity score=0): £390 for all elective admissions and £590 for all other admissions. The children were assigned the IMD decile of their LSOA of birth, ignoring any subsequent movements.

## Results

The 2003/2004 final analysis cohort consisted of 515 889 baby records, 91% of the unique birth records identified. For the 2013/2014 cohort, we obtained the IMD decile for 576 624 (91%) of the 634 293 identified baby records. Both cohorts were representative of the national population in terms of IMD decile, maternal age, birth weight and gestation.^16–18^ Further details can be found in the online supplemental appendix.

**Figure 1:**
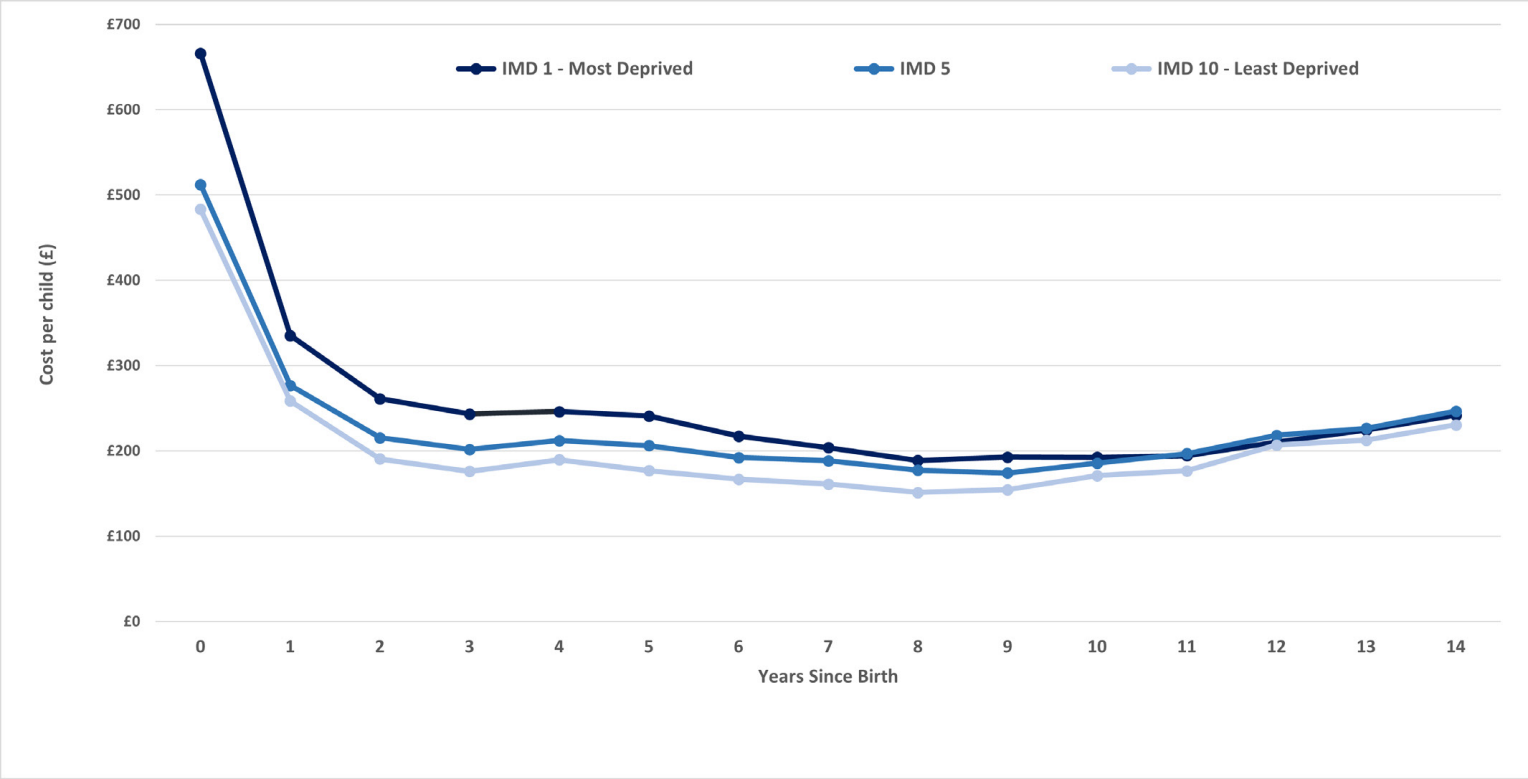
2003 cohort, cost per child (admissions and outpatient attendances) per year by IMD decile.

For the 2003/2004 cohort, over the first 15 years of life, those born in the most deprived areas had the highest levels of hospital utilisation. The highest annual cost was observed in the year following birth, which was also where the largest absolute difference between the most and least deprived deciles was observed. On average, in the first year of life, hospital use (outpatient and admitted) for each child from the most deprived decile cost over 38% more than the children from the least deprived areas (£666 vs £483) (figure 1).

**Figure 2:**
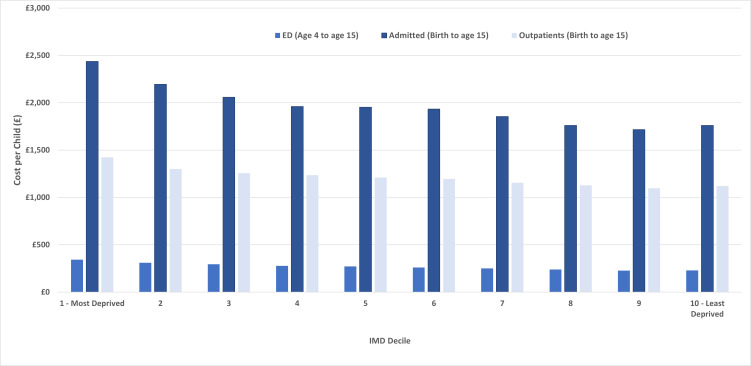
2003 cohort, total hospital costs to age 15 years by IMD decile

Across all groups, the highest costs were accrued for inpatient admissions, followed by outpatient attendances and then ED visits. Up to the age of 15 years, the difference between the most and least deprived deciles for admissions was £676, 38% higher, and for outpatients £305, 27% higher. This is a combined difference of £981, 34% higher, for those born in the most deprived areas compared with the least deprived areas. From the age of 4–15 years, ED costs were £113, 49% higher for those in the most deprived decile compared with the least deprived decile (table 1, figure 2).

**Table 1:** Admitted, outpatient and A and E utilisation and costs per child up to age 15 years by IMD decile.

IMD	Children in cohort (n=515 889)		Average number of admissions/attendances			Average costs (SD) per child (95% CI)		
	N	%	Emergency department attendances* (age 4–15 years)	Inpatient admissions(birth to age 15 years)	Outpatient attendances (birth to age 15 years)	Emergency department attendances * (age 4–15 years)	Inpatient admissions (birth to age 15 years)	Outpatient attendances (birth to age 15 years)
1 Most deprived	77 326	15.0%	3.2	2.0	10.6	£342 (447) (£339 to £345)	£2436 (7660) (£2382 to £2490)	£1424 (2621) (£1406 to £1442)
2	65 911	12.8%	2.9	1.7	9.7	£309 (419) (£306 to £312)	£2194 (7320) (£2138 to £2250)	£1300 (2437) (£1281 to £1319)
3	57 830	11.2%	2.8	1.6	9.4	£294 (405) (£291 to £297)	£2058 (7127) (£2000 to £2116)	£1257 (2337) (£1238 to £1276)
4	51 515	10.0%	2.6	1.6	9.3	£277 (375) (£274 to £280)	£1961 (6256) (£1907 to £2015)	£1236 (2451) (£1215 to £1257)
5	48 051	9.3%	2.6	1.6	9.1	£271 (366) (£268 to £274)	£1952 (6378) (£1895 to £2009)	£1210 (2205) (£1190 to £1230)
6	45 581	8.8%	2.5	1.5	8.9	£260 (356) (£257 to £263)	£1934 (6944) (£1870 to £1998)	£1194 (2262) (£1173 to £1215)
7	43 295	8.4%	2.4	1.5	8.6	£250 (342) (£247 to £253)	£1854 (6743) (£1790 to £1918)	£1155 (2182) (£1134 to £1176)
8	42 992	8.3%	2.2	1.4	8.4	£238 (336) (£235 to £241)	£1761 (5624) (£1708 to £1814)	£1127 (2158) (£1107 to £1147)
9	42 497	8.2%	2.1	1.3	8.2	£226 (313) (£223 to £229)	£1717 (5880) (£1661 to £1773)	£1096 (2105) (£1076 to £1116)
10 Least deprived	40 891	7.9%	2.2	1.4	8.4	£229 (309) (£226 to £232)	£1760 (6240) (£1700 to £1820)	£1119 (2128) (£1098 to £1140)
Cost difference between most and least deprived (95% CI)						£113 (£109 to £118)	£676 (£595 to £757)	£305 (£277 to £333)

*Emergency department (ED) data were unavailable prior to 2007/2008, so ED data start from age 4 years.

IMD, Index of Multiple Deprivation.

**Figure 3:**
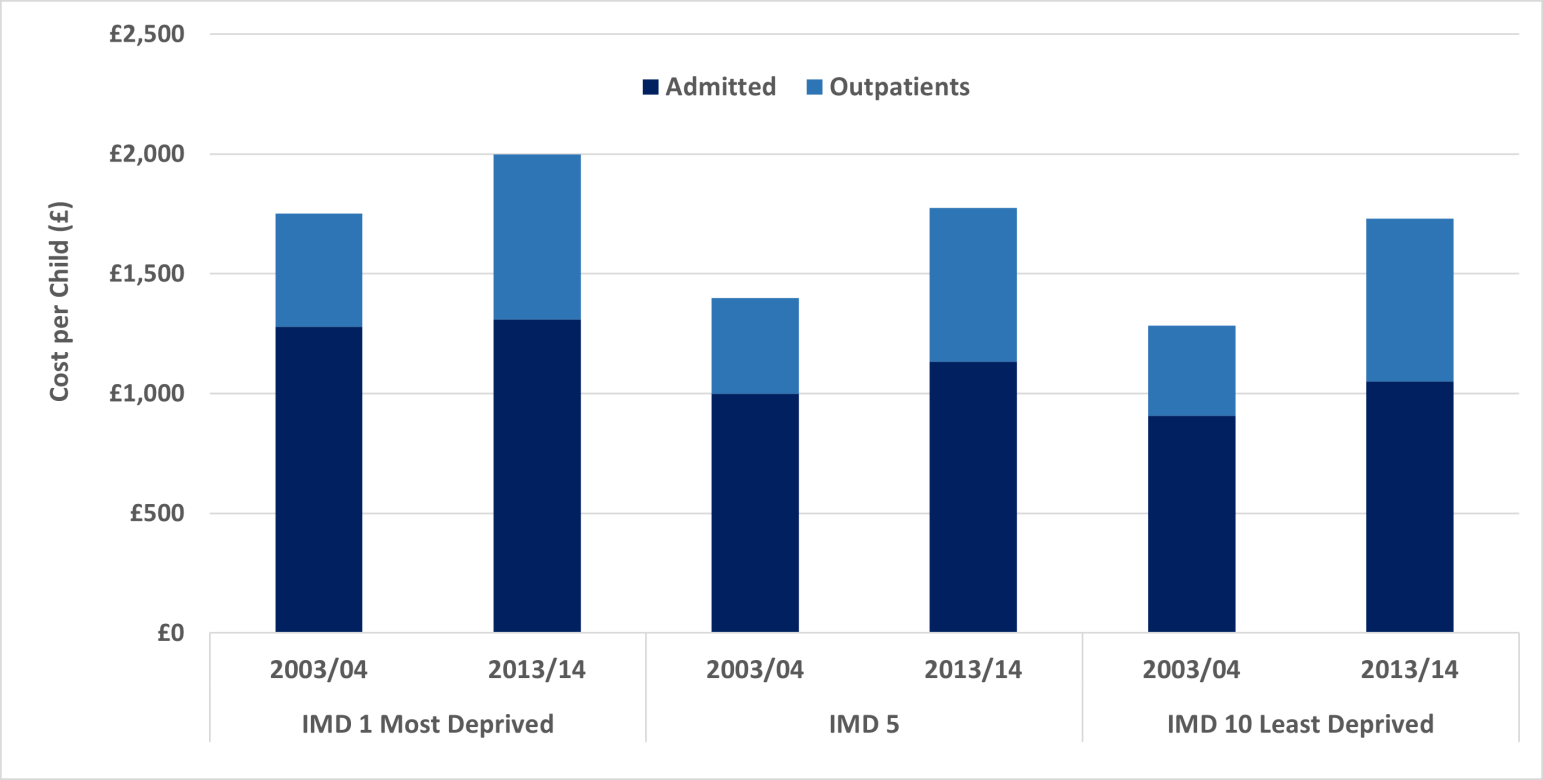
Comparison of cohorts. Admitted and outpatient costs per child up to age 5 years by IMD decile.

There are significant shifts in utilisation patterns between the 2003 and 2013 cohorts. Across all deprivation deciles, hospital costs have increased in real terms from the 2003 to the 2013 cohort, while the difference between the most and least deprived has decreased, for admissions from £371 in 2003 to £210 in 2013 and for outpatients from £97 to £10 (table 2, figure 3).

**Table 2:** Admitted and outpatient costs per child up to age five by IMD decile

IMD	n=515 889	n=576 624	Inpatient admissions		Outpatient attendances	
			Average costs (SD) per child (95% CI)		Average costs (SD) per child (95% CI)	
	2003	2013	2003	2013	2003	2013
1 Most deprived	77 326	82 804	£1279 (4280) (£1249 to £1309)	£1310 (3607) (£1285 to £1335)	£473 (944) (£466 to £480)	£689 (1477) (£679 to £699)
2	65 911	76 282	£1158 (4385) (£1125 to £1191)	£1217 (4475) (£1185 to £1249)	£436 (907) (£429 to £443)	£656 (1416) (£646 to £666)
3	57 830	68 773	£1090 (4219) (£1056 to £1124)	£1196 (3672) (£1169 to £1223)	£421 (899) (£414 to £428)	£656 (1356) (£646 to £666)
4	51 515	62 742	£1024 (3712) (£992 to £1056)	£1162 (3612) (£1134 to £1190)	£413 (910) (£405 to £421)	£651 (1407) (£640 to £662)
5	48 051	56 752	£999 (3574) (£967 to £1031)	£1133 (3274) (£1106 to £1160)	£400 (831) (£393 to £407)	£642 (1345) (£631 to £653)
6	45 581	51 313	£989 (4038) (£952 to £1026)	£1078 (3513) (£1048 to £1108)	£397 (850) (£389 to £405)	£644 (1317) (£633 to £655)
7	43 295	47 083	£966 (3955) (£929 to £1003)	£1059 (3424) (£1028 to £1090)	£382 (804) (£374 to £390)	£642 (1273) (£631 to £653)
8	42 992	44 704	£924 (3670) (£889 to £959)	£1077 (3124) (£1048 to £1106)	£376 (823) (£368 to £384)	£660 (1367) (£647 to £673)
9	42 497	44 429	£898 (3890) (£861 to £935)	£1048 (3286) (£1017 to £1079)	£368 (803) (£360 to £376)	£656 (1368) (£643 to £669)
10 Least deprived	40 891	41 742	£908 (3726) (£872 to £944)	£1050 (5193) (£1000 to £1100)	£376 (805) (£368 to £384)	£679 (1418) (£665 to £693)
Cost difference between most and least deprived (95% CI)			£371 (£324 to £419)	£260 (£215 to £304)	£97 (£86 to £107)	£10 (-£6 to £26)

IMD, Index of Multiple Deprivation.

## Discussion

Children born to mothers living in the most deprived areas consistently had higher hospital use and costs than those born in less deprived areas. Over the course of their childhood (to age 15 years), children’s use of inpatient and outpatient hospital services costs the NHS £2879 per child from the least deprived areas and, 34% more, £3860 per child born into the most deprived areas. The difference was most marked in the first year of life, where children born to mothers in the most deprived areas cost 38% more in inpatient and outpatient hospital utilisation than those in the least deprived areas. The cost of ED attendances is not available for the whole length of the cohort, but between the ages of 4 and 15 years, ED attendances for children born in the most deprived areas cost, on average, £342 (95% CI £339 to £345) compared with those in the least deprived areas (£229, 95% CI £226 to £232)—a difference of over 49%.

Although we are not aware of other studies measuring the long-term hospital costs of children born into relative deprivation, our findings are consistent with the extensive literature on the association between childhood deprivation and poorer health. Previous research also found that more deprived children and young people are more likely to go to hospital EDs and be admitted to a hospital as an emergency and that the gap between the most and least deprived narrowed slightly between 2005/2006 and 2015/2016.^9^ Another study found that children’s use of hospital urgent and outpatient care rose between 2007 and 2017, while GP consultation rates fell.^19^ These papers are consistent with our comparison of the cohorts for the first 6 years of life. The average cost per child increased from 2003 to 2013, with outpatient attendances accounting for an increasing proportion of overall utilisation and costs per child.

### Strengths and limitations

Our analysis uses routinely collected administrative data to illustrate patterns of hospital utilisation and costs, some of which are potentially amenable to policy intervention. Deprivation is associated with poorer health in childhood,^3^ poorer health in later life^5^ and greater use of NHS services.^20^ Using administrative data like HES for research purposes has many strengths, including generating a large sample size (including individuals who may be less likely to participate in primary data collection), little loss to follow-up and generating evidence with high external validity and policy applicability.^21^ It does, however, have limitations, including missing data—which can result from poor recording, from individuals not seeking care or from insufficient information to enable data linkage.^21^

There are some limitations to our analyses. First, deprivation was assigned using the LSOA of residence of the mother at the time of birth and the corresponding area-level deprivation score, rather than any direct measure of the socioeconomic status of the actual household. Nevertheless, area-level deprivation indicators have been demonstrated to be associated strongly with all the main domains of deprivation at individual level, including health.^22^

Second, our analysis is restricted to hospital-based care (inpatient admissions, outpatient and ED attendances). We did not attempt to include the costs of using primary care, partly because of data shortages but also because primary care access can differ markedly between areas, and this is likely to translate into differences in utilisation which result from differences in the availability of appointments rather than reflecting underlying patient need. Nussbaum and colleagues^23^ showed using data from 2015 to 2020 that there were 1.4 fewer full-time equivalent GPs per 10 000 patients in the most deprived areas compared with the least deprived. It is also important to note that other health systems may provide a higher proportion of outpatient care in ambulatory settings, and that our results may not be generalisable to different contexts.

Third, although we made every effort to assign an LSOA to each baby, using both deterministic and probabilistic methods of linking mother and baby data, around 9% of babies in each cohort did not have a matched LSOA. The variables used for linkage are included in the maternity tail; this is obtained from information entered into local maternity databases and was not mandated to return to NHS digital.^14^ This leads to a large variation between hospital sites in terms of data completeness and quality which impacts the number of mother and baby records linked from individual sites. However, overall, both cohorts were representative of the national population in terms of IMD decile, maternal age, birth weight and gestation (online supplemental appendix).

Fourth, we could not use actual prices paid because payment based on HRGs was gradually rolled out in the NHS from 2003/2004 onwards and did not cover all inpatient care until several years into our study.^24^ Our approach of assigning average inpatient costs by treatment area (HRG chapter) underestimates high-cost, complex cases. If these types of cases are more prevalent in children living in deprived areas, this will bias the cost-deprivation gradient towards uniformity.

Finally, nationwide data on ED attendances were not available before 2007, so we were only able to calculate these costs from age 4 to 15 years.

### Implications for policy and practice

Reducing health inequalities was made a duty for the Secretary of State in the Health and Social Care Act 2012. The Academy of Medical Royal Colleges, the Royal College of Paediatrics and Child Health (RCPCH) and the Faculty of Public Health all prioritise health inequalities among children and young people and the need for prevention.^25^ This study highlights the differential use of hospital services for children born into relative deprivation and their higher costs, confirming the importance of addressing inequalities at all levels of policy and practice. As well as implementing high-level policies to improve the social determinants of health targets, for example, setting a target to end child poverty (as recommended by the RCPCH),^26^ there may be a role for more local and regional interventions to improve access to primary and community services in deprived areas^4^ and to invest in education and community building to build neighbourhood resilience.^1^

## Conclusion

Hospital utilisation and associated costs were consistently higher for children born into more deprived areas over the first 15 years of life, particularly in the first year. On average, children born to mothers living in the most deprived areas, use inpatient and outpatient hospital services which cost 38% more in the first year of life and 34% more over the course of their childhood.

## Data Availability

Data may be obtained from a third party and are not publicly available. Hospital Episode Statistics (HES) data can be accessed via NHS Digital (https://digital.nhs.uk/data-and-information/data-tools-and-services/data-services/hospital-episode-statistics). Applications to access HES data can be made directly to NHS digital. The authors do not have permission to share patient-level HES data.
