# Supplementary material for: Examining the hospital costs of children born into relative deprivation in England
Source: J Epidemiol Community Health. 2024 May 15;78(8):493–9. doi: 10.1136/jech-2023-221175 (PMC11287521; doi:10.1136/jech-2023-221175)
Supplement: Supplementary data [file jech-2023-221175supp001.pdf]

Appendix

Table 1 - Number of records linked using each method

		**2003/04 Cohort N=567,347**		**2013/14 N=634,293**	
		**N**	**% of Cohort**	**N**	**% of Cohort**
Deterministically linked		271,111		413,870	
	Valid IMD	269,719	47.5	410,870	64.8
Probabilistically linked		210,011			
	Valid IMD	209,020	36.8	-	-
IMD – baby		37,150	6.5	165,754	26.1
**Final**		**515,889**	**90.9**	**576,624**	**90.9**

Table 2 - Linking Variables

**Variable**		**Deterministic**	**Probabilistic**
**Provider Code**	**procode**	✓	✓
**GP practice**	**gpprac**	✓	✓
**Maternal Age**	**matage**	✓	✓
**Birth Weight**	**birweit**	✓	✓
**Gestation**	**gestat**	✓	✓
**Birth Order**	**birordr**	✓	✓
**Baby Sex**	**babysex**	✓	✓
**Estimated delivery date**	**opdte/birthdate**	**X**	✓
**First Antenatal Assessment data**	**anasdate**	**X**	✓
**Gestation at first antenatal assessment**	**anagest**	**X**	✓
**Delivery method**	**delmeth**	**X**	✓
**Intended delivery place**	**delinten**	**X**	✓
**Actual Delivery place**	**delplac**	**X**	✓
**Method to induce labour**	**delonset**	**X**	✓
**Anaesthetic given during labour/delivery**	**delprean**	**X**	✓
**Anaesthetic given post labour/delivery**	**delposan**	**X**	✓
**Status person conducting delivery**	**delstat**	**X**	✓
**Birth status**	**birstat**	**X**	✓
**Resuscitation method**	**biresus**	**X**	✓
**Ethnic category**	**ethnos**	**X**	✓
**Postcode district**	**postdist**	**X**	✓
**Episode start Date**	**epistart**	**X**	✓
**Episode end Date**	**epidend**	**X**	✓

Table 3 - Completion rate of variables of important linking variables

	2003			2013		
	**All N=576,347**	**Included N=515,889**	**Not Included N=51,458**	**All N=634,293**	**Included N=576,624**	**Not Included N=57,669**
**Provider Code**	**100%**	100%	100%	100%	100%	100%
**GP Practice**	**99.7%**	99.7%	99.7%	99.9%	99.9%	98.9%
**Maternal age**	**61.4%**	64.7%	28.7%	66.3%	71.1%	18.8%
**Birth Weight**	**58.3%**	60.8%	32.7%	82.4%	82.6%	80.3%
**Gestation**	**55.6%**	58.6%	26.6%	76.7%	79.8%	45.2%
**Birth Order**	**65.7%**	68.5%	37.4%	81.9%	82.7%	73.8%
**Baby Sex**	**99.9%**	99.9%	99.9%	99.9%	99.9%	99.9%

Table 4 – Cohort Characteristics

		2003		2013	
		**Included N=515,889**	**Not Included N=51,458**	**Included N=576,624**	**Not Included N=57,669**
**Maternal age (years)**	**Valid N**	333,851	14,745	409,701	10,827
	**Mean (SD)**	29.3 (6.0)	28.8 (6.0)	29.5 (5.8)	30.1(5.9)
**Gestation (weeks)**	**Valid N**	302,030	13,669	460,297	26,069
	**Mean (SD)**	39.1 (2.5)	38.6 (3.0)	39.0 (2.4)	39.0 (2.4)
**Birth Weight (g)**	**Valid N**	313,723	16,827	476,423	46,277
	**Mean (SD)**	3297.5 (652.2)	3130.3 (832.5)	3338.7 (597.3)	3313.6 (604.1)
**Ethnicity**	**White**	250,062 (48%)	27,912 (54%)	407,586 (71%)	35,079 (61%)
	**Black**	13,576 (3%)	1,438 (3%)	23,703 (4%)	2789 (5%)
	**Asian**	27,488 (5%)	2,147 (4%)	60,993 (11%)	6702 (12%)
	**Mixed**	8,384 (2%)	1,062 (2%)	26,506 (5%)	2951 (5%)
	**Other**	12,723 (2%)	1,383 (3%)	20,383 (3.8%)	2057 (4%)
	**Missing**	203,656 (39%)	17516 (34%)	37,453(6%)	8091 (14%)
**Region of Hospital**	**North East**	25,974 (5%)	549 (1%)	29,812 (5%)	50 (<1%)
	**North West**	72,749 (14%)	2,403 (5%)	76,114 (13%)	5,190 (9%)
	**Yorkshire and the Humber**	55,564 (11%)	1,924 (4%)	58,455 (10%)	2,373 (4%)
	**East Midlands**	31,911 (6%)	9,109 (18%)	37,836 (7%)	6,510 (11%)
	**West Midlands**	60,748 (12%)	3,801 (7%)	55,208 (10%)	15,051 (26%)
	**East of England**	44,699 (9%)	14,048 (27%)	59,894 (10%)	7,347 (13%)
	**London**	98,439 (19%)	11,057 (21%)	113,207 (20%)	13,805 (24%)
	**South East**	79,789 (15%)	6,969 (13%)	90,385 (16%)	6,933 (12%)
	**South West**	46,016 (9%)	1,598 (3%)	55,713 (10%)	410 (1%)

Table 5 - Comparison to ONS National Statistics

		2003		2013	
		ONS	Cohort	ONS	Cohort
**Maternal age (Years)**	**Mean**	29.4	29.3	30.0	29.5
**Birth Weight(g)**	**Under 1500**	1.3 %	1.9 %	1.1%	1.1%
	**1500-1999**	1.6 %	1.4 %	1.4%	1.4%
	**2000 – 2499**	4.8 %	4.7 %	4.6%	4.6%
	**2500-2999**	17.2 %	17.4 %	16.3%	16.6%
	**3000-3499**	35.6 %	35.7 %	35.3%	35.7%
	**3500+**	39.3 %	38.9 %	40.3%	40.7%
**IMD**	**1 – Most deprived**	14.6%	15.0%	14.3%	14.4%
	**2**	12.4%	12.8%	13.0%	13.2%
	**3**	10.9%	11.2%	12.0%	11.9%
	**4**	9.9%	10.0%	10.8%	10.9%
	**5**	9.3%	9.3%	9.6%	9.8%
	**6**	8.8%	8.8%	9.2%	8.9%
	**7**	8.5%	8.4%	8.3%	8.2%
	**8**	8.5%	8.3%	8.1%	7.8%
	**9**	8.5%	8.2%	7.7%	7.7%
	**10 – Least Deprived**	8.5%	7.9%	6.9%	7.2%

ONS data obtained from ONS website

ONS. Number of live births by Index of Multiple Deprivation (IMD), 2003 to 2020, England 2022 [Available from: https://www.ons.gov.uk/peoplepopulationandcommunity/birthsdeathsandmarrages/livebirths/adhocs/14333numberoflivebirthsbyindexofmultipledeprivationimd2003to2020england.

ONS. Birth statistics. Review of the Registrar General on births and patterns of family building in England and Wales, 2003 2004 [Available from: https://webarchive.nationalarchives.gov.uk/ukgwa/20150910114054/http://www.ons.gov.uk/ons/publications/re-reference-tables.html?edition=tcm%3A77-156297.

ONS. Characteristics of Birth 1, England and Wales, 2013 Edition 2014 [Available from: https://www.ons.gov.uk/peoplepopulationandcommunity/birthsdeathsandmarriages/livebirths/datasets/characteristicsofbirth1englandandwales.
